# Serial pseudoprogression of metastatic malignant melanoma in a patient treated with nivolumab: a case report

**DOI:** 10.1186/s12885-017-3785-4

**Published:** 2017-11-21

**Authors:** Yukinori Ozaki, Junichi Shindoh, Yuji Miura, Hiromichi Nakajima, Ryosuke Oki, Miyuki Uchiyama, Jun Masuda, Keiichi Kinowaki, Chihiro Kondoh, Yuko Tanabe, Tsuyoshi Tanaka, Shusuke Haruta, Masaki Ueno, Shigehisa Kitano, Takeshi Fujii, Harushi Udagawa, Toshimi Takano

**Affiliations:** 1 Department of Medical Oncology, Toranomon Hospital, 2-2-2 Toranomon Minato-ku, Tokyo, 105-8470 Japan; 2 Hepatobiliary-pancreatic Surgery Division, Department of Digestive Surgery, Toranomon Hospital, Tokyo, Japan; 3 Okinaka Memorial Institute for Medical Disease, Tokyo, Japan; 4 Department of Pathology, Toranomon Hospital, Tokyo, Japan; 5 Department of Gastroenterological Surgery, Toranomon Hospital, Tokyo, Japan; 6 Department of Experimental Therapeutics, Exploratory Oncology Research and Clinical Trial Center, National Cancer Center, Tokyo, Tsukiji Japan

**Keywords:** Esophageal malignant melanoma, Nivolumab, Pseudoprogression, Serial

## Abstract

**Background:**

Pseudoprogression refers to a specific pattern of response sometimes observed in malignant melanoma patients receiving treatment with immune-checkpoint inhibitors. Although cases with pseudoprogression documented once have been reported previously, there have been no case reports yet of pseudoprogression events documented twice during treatment.

**Case presentation:**

A 55-year-old man underwent surgery for locally advanced esophageal malignant melanoma and received postoperative adjuvant interferon therapy. However, he presented with multiple liver and bone metastases at 6 months after the surgery, and was initiated on treatment with nivolumab 2 mg/kg every 3 weeks as the first-line treatment for recurrent disease. Follow-up computed tomography revealed that the liver metastases initially increased transiently in size, but eventually regressed. However, while the liver metastases continued to shrink, a new peritoneal nodule emerged, that also subsequently shrinked during the course of treatment with nivolumab. With only grade 1 pruritus, the patient continues to be on nivolumab treatment at 15 months after the induction therapy, with no progression observed after the second episode of pseudoprogression in the liver and peritoneal nodule.

**Conclusions:**

We present the case of a patient with metastatic malignant melanoma who showed the unique response pattern of serial pseudoprogression during treatment with nivolumab. This case serves to highlight the fact that development of a new lesion may not always signify failure of disease control during treatment with nivolumab.

## Background

The global incidence of malignant melanoma continues to rise, and unresectable or metastatic melanoma has a poor prognosis, with a reported median survival time of 6–8 months [1–4]. Currently, immunotherapy is the standard therapy for unresectable or metastatic melanoma, and the prognosis of patients with cutaneous metastatic melanoma has improved. Treatment with ipilimumab, an antibody directed against cytotoxic T-lymphocyte antigen-4, has been demonstrated to yield long-term survival in approximately 20% of patients with advanced melanoma [5, 6]. Nivolumab is a fully humanized IgG4 programmed death 1 (PD-1) immune-checkpoint inhibitor antibody, and PD-1 is expressed on antigen-stimulated T cells and tumor cells. Interaction of PD-1 with its ligands inhibits the antitumor activity of the cytotoxic T cells. Nivolumab blocks the interaction between the PD-1 receptor and the programmed death ligands, PD-L1 and PD-L2, and disrupts the negative signal that regulates T-cell activation and proliferation. Nivolumab treatment has been shown to yield a more favorable survival benefit in previously untreated patients with metastatic melanoma not harboring a *BRAF* mutation as compared to dacarbazine, and it has been approved in Japan for the treatment of unresectable or metastatic melanoma [7]. According to a previous report, a durable response was achieved with nivolumab in approximately 40% of patients with cutaneous metastatic melanoma [8].

Mucosal melanoma is rare, accounting for 2% or less of all cases of melanoma, and the prognosis of mucosal metastatic melanoma is poor, with a 5-year survival rate of less than that reported for cutaneous or uveal melanoma [9–11]. It has also been reported that mucosal melanoma is an aggressive subtype of melanoma that is resistant to immune checkpoint inhibitors, and that patients with this disease show lower response rates to treatment and shorter survival [12].

With immunotherapy becoming increasingly easily available to patients, a major problem that has arisen is the lack of an accurate method yet to determine the clinical efficacy of immunomodulatory drugs. Recently, immune-related patterns of response, which cannot be evaluated by the Response Evaluation Criteria In Solid Tumors (RECIST) have been reported in some studies. According to one study, 4% of patients with metastatic melanoma receiving treatment with nivolumab experienced pseudoprogression [13]. Another study classified pseudoprogression into early and delayed pseudoprogression [14]. Nevertheless, the precise details of the patterns of response to immunotherapy remain unclear. Herein, we report the first case that experienced pseudoprogression twice in a patient with metastatic malignant melanoma, who responded to treatment with nivolumab for over 1 year.

## Case presentation

**Fig. 1:**
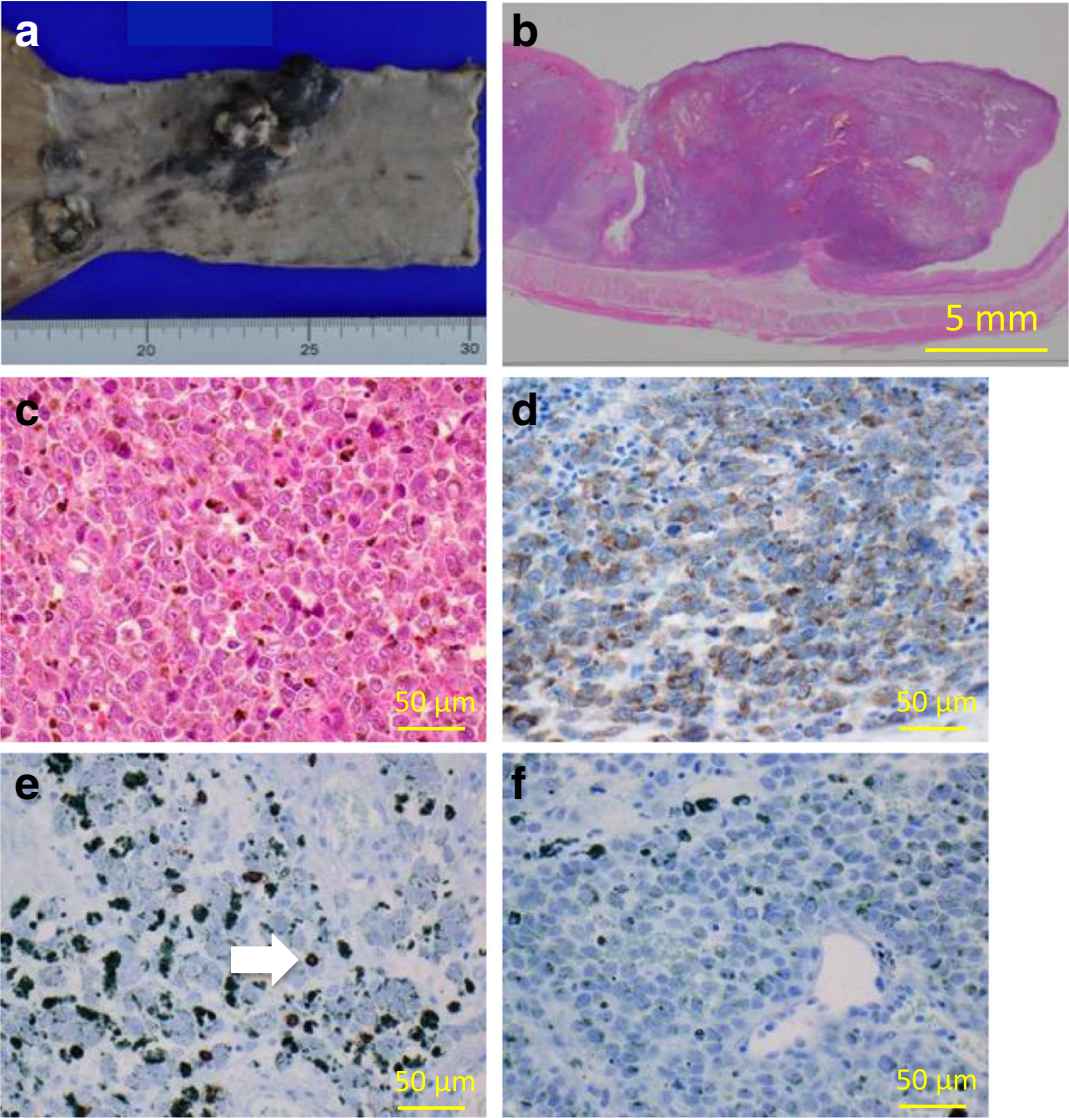
The gross and histological findings of the resected esophageal melanoma. **a** Gross examination. There was an irregular elevated black lesion in the lower esophagus. **b** Macroscopic findings (Hematoxylin-eosin staining). The tumor showed proliferation of eosinophilic tumor cells. **c** Hematoxylin-eosin staining of tumor cells. There were solid proliferation and tumor cells had large round nuclear. Melanin pigments were sometimes found. **d** Melan A immunostaining and Giemsa as counterstain. Tumor cells were diffusely positive and there were some of melanin pigments. **e** CD8 immunostaining and Giemsa as counterstain. Few numbers of cells were positive (arrowhead) and melanin pigments were seen. **f** PD-L1/CD274 (SP142) immunostaining and Giemsa as counterstain. There were no positive cells in the tumor. Melanin pigments were found

A 55-year-old previously healthy man was detected as having an abnormal endoscopic finding in an organized gastric cancer screening examination conducted in July 2014. He was afebrile and other vital signs were normal. Physical examination revealed no abnormalities. Fiberoptic gastrointestinal endoscopy showed a 20-mm black elevated lesion in the middle-third of the intrathoracic esophagus. Enhanced computed tomography (CT) revealed nodular wall thickening measuring 15 × 10 mm in size in the middle-third of the intrathoracic esophagus, with no significant lymph node or distant metastasis. Esophageal biopsy was performed and showed proliferation of large round tumor cells and melanophages. Immunohistochemically, these round cells were diffusely positive for human melanin black 45 (HMB45) (diluted 1/10 dilution; Leica, Wetzlar, Germany) and melan A (1/1000 dilution; Thermo Fisher Scientific, Waltham, MA) and partly positive for S-100 protein (1/1000 dilution; Dako, Glostrup, Denmark). There were no expression of BRAF V600E (1/500 dilution; Spring Bioscience, Pleasanton, CA, USA) in tumor cells, and Ki67 (1/1 dilution; Roche, Basel, Switzerland) labelling index of them was 20%. On the basis of these findings, the patient was diagnosed as an esophageal malignant melanoma, clinical T4aN0M0 (stage IVA, UICC, 7th Edition) and was treated in August 2014 by video-assisted thoracic esophagectomy, proximal gastrectomy and 3-field lymph node dissection with ileocolic reconstruction. Macroscopically, the tumor was an irregular elevated black mass of 60 × 25 mm in size that was consistent with the endoscopic findings (Fig. 1a). A microscopic examination demonstrated that the tumor was located in the submucosal lesion and that there were solid proliferation of eosinophilic tumor cells without tubular or papillary structures (Fig. 1b and c). Tumor cells had large round nuclear and melanin pigments were sometimes found in the cytoplasm of tumor cells. Immunohistochemical staining for HMB45 and melan A was positive in tumor cells as with the biopsy specimen (Fig. 1d). Based on these morphological features and immunohistochemical findings, the tumor was diagnosed as a malignant melanoma in the esophagus with T3 invasion, node-positive (3/100), and the disease stage was classified as pT3N1M0 stage III (UICC, 7th Edition). Immunohistochemically, few numbers of cells were positive for CD8 (1/1 dilution; Roche) (Fig. 1e) and PD-L1/CD274 (clone SP142, 1/50 dilution; Spring Bioscience) expression was <1% in tumor-infiltrating immune cells and tumor cells (Fig. 1f).

**Fig. 2:**
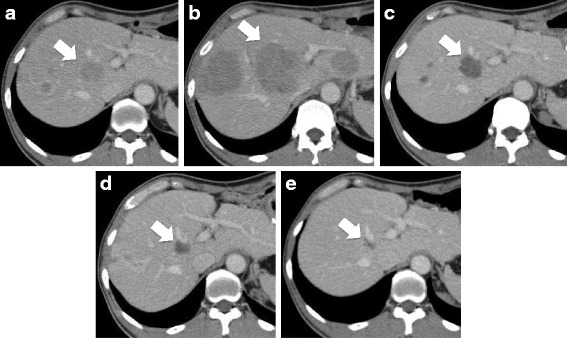
Liver metastases from malignant melanoma which showed early pseudoprogression during treatment with nivolumab. **a** Liver metastases (arrowhead indicates the target lesion: 31 mm) before the start of treatment with nivolumab. **b** CT at the 3-month assessment: the liver metastases had increased in size (target lesion: 63 mm). **c** CT at the 5-month assessment: the liver metastases had shrunk (target lesion: 31 mm), associated with a change of the density. **d** CT at the 8-month assessment: the target lesion was 20 mm in diameter. **e** CT at the 12-month assessment: the target lesion was 13 mm in diameter

**Fig. 3:**
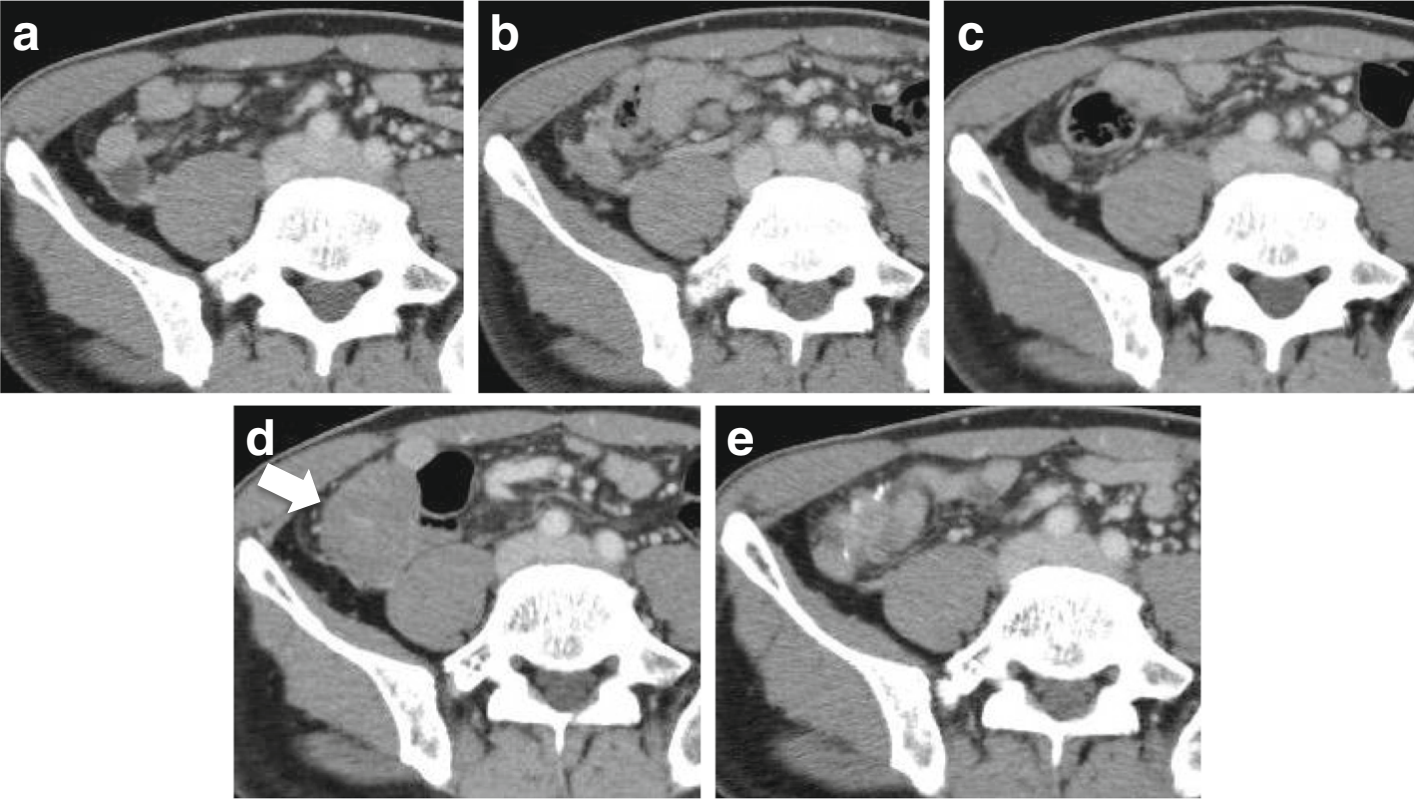
Peritoneal nodule emerged in the abdomen. **a**, **b**, **c** No tumor lesion was detected before the start of treatment with nivolumab (**a**), at the 3-month (**b**) and 5-month assessment (**c**). **d** At the 8-month assessment, a new peritoneal nodule was detected in the abdomen (arrowhead). The nodule measured 44 × 21 mm in size. **e** At the 12-month assessment, the nodule had decreased in size and an opacity could be seen next to the intestinal tract

**Fig. 4:**
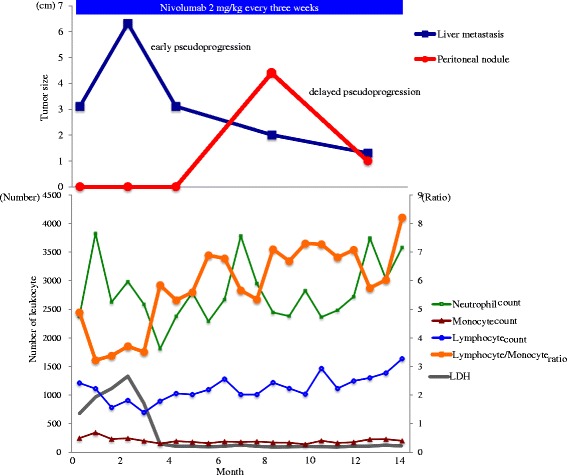
Changes of the tumor lesions and number of leukocytes. The upper graph shows the changes in the sizes of the target lesions. The liver metastasis showed a transient increase in size at the first assessment (3 months), whereas at the next assessment (5 months) it had shrunk in size, fulfilling the definition of early pseudoprogression. A peritoneal nodule emerged in the CT obtained at the 8-month assessment, but the nodule had regressed at the 12-month assessment (delayed pseudoprogression). The lower graph shows the absolute number of neutrophils, monocytes, monocyte and the serum level of lactate dehydrogenase (LDH). The count of lymphocytes showed a tendency towards increase, while that of monocytes was stable, resulting in elevation of the lymphocyte/monocyte ratio

Although the patient received postoperative adjuvant interferon treatment, a follow-up CT obtained at 6 months after the surgery revealed multiple liver metastases (Fig. 2a). Positron emission tomography (PET)-CT revealed multiple bone metastases, although the patient had no symptoms. The physical examination and blood test findings were normal, except for elevation of the serum lactate dehydrogenase (LDH) level to 711 IU/L. The patient was diagnosed as having recurrent malignant melanoma with liver and bone metastases, and was started on treatment with nivolumab at 2 mg/kg every 3 weeks as the first-line treatment for the recurrent disease, and denosumab as treatment for the bone metastases in February 2015. The liver metastases showed an initial transient increase in size (+119%) in the CT obtained at the 3-month assessment (Fig. 2b), whereas at the 5-month assessment, CT showed shrinkage of the liver metastases with a change of their density (Fig. 2c), fulfilling the definition of early pseudoprogression. While the liver metastases continued to shrink (Fig. 2d and e), a new peritoneal nodule emerged in the abdomen that was detected in the CT obtained at the 8-month assessment (Fig. 3a-d). The peritoneal nodule was 44 × 21 mm in size (Fig. 3d), whereas at the 12-month assessment, the nodule was found to have regressed (Fig. 3e) (delayed pseudoprogression). The changes in the largest diameter of the tumor lesions are shown in Fig. 4. No tumor recurrence or progression has been observed after the second episode of pseudoprogression in the liver, peritoneal nodule and bone. Blood tests showed transient elevation of the serum LDH to 1351 IU/L at 1 month after the initiation of nivolumab treatment, with the levels returning to normal range by 4 months after the start of treatment. During the treatment, the absolute number of lymphocyte tended to increase and that of monocyte was stable, resulting the elevation of the ratio of lymphocyte/monocyte. Grade 1 pruritus was the only adverse event observed. No diarrhea or signs of intraperitoneal infection were observed during the treatment. At the time of the follow-up in May 2016, he had received nivolumab treatment for 15 months and had shown, until then, no further signs of clinical disease progression.

## Discussion and conclusions

Here we report the patient presenting recurrent malignant melanoma with liver and bone metastases who received nivolumab treatment. During the treatment, the liver metastases showed an initial transient increase and subsequent shrinkage, which meets with the criteria of early pseudoprogression. After the pseudoprogression in liver metastases, a new peritoneal lesion emerged and it regressed thereafter. Although pseudoprogression caused by nivolumab treatment is reported in about 4% of patients with metastatic melanoma [13], this unique serial response pattern has never been reported so far.

Immunotherapeutic agents, such as nivolumab, ipilimumab and pembrolizumab, are being tested as novel anticancer therapies for many types of solid tumors. Because of the novel mechanisms of action, immune-related patterns of response that cannot be evaluated by conventional RECIST have been reported, and the landscape of the patterns of response in solid tumors treated with immunotherapeutic agents remains unclear. One study classified pseudoprogression into early and delayed pseudoprogression [14]. Early pseudoprogression is defined as a ≥ 25% increase of the tumor burden or a new lesion detected at imaging assessment at 12 weeks, which, however, is not confirmed as progressive disease according to Immune-related Response Criteria (irRC) at the next assessment. Delayed pseudoprogression is defined as a ≥ 25% increase of the tumor burden or a new lesion at any imaging assessment performed after the first imaging assessment, which is not confirmed as progressive disease according to the irRC at the subsequent imaging assessment. Another study showed that 8.9% of metastatic melanoma patients treated with ipilimumab experienced pseudoprogression, classified as early pseudoprogression (4.6%) or delayed pseudoprogression (4.3%) [15]. In the KEYNOTE-001 melanoma expansion cohorts, early pseudoprogression was observed in 4.6% patients and delayed pseudoprogression in 2.8% patients who had received treatment with pembrolizumab [14]. However, there has been no report on serial patterns of pseudoprogression observed in one patient with malignant melanoma. Development of new lesions after early pseudoprogression could be evaluated as actual disease progression and mistakenly lead to discontinuation of treatment in patients who would potentially benefit from treatment continuation like present case.

The treatment response pattern of pseudoprogression is sometimes encountered in patients with melanoma receiving immunotherapy. However, the mechanism of pseudoprogression is not yet clearly understood. Therefore, it is especially difficult for us to explain the mechanism of pseudoprogression events occurring twice in the current patient. Different mechanisms could have been operative between the early pseudoprogression and delayed pseudoprogression in this case. The early pseudoprogression was detected in the liver, where metastases were already present before the treatment was started. This response in the liver could possibly be explained by inflammatory cell infiltration or necrosis, as suggested previously [14]. On the other hand, we would like to speculate on some possible mechanisms underlying the delayed pseudoprogression. First, the heterogeneity of the immune microenvironments between the liver metastases and peritoneal nodules might have been responsible for the pseudoprogression events detected at different time-points. Several studies have reported the existence of a correlation between the clinical response and expression of PD-L1 on the tumor cells [16]. Immunohistochemically, the resected primary esophageal tumor showed no PD-L1 expression (<1%) in tumor-infiltrating immune cells and tumor cells, and it suggests no PD-L1 expression originally in the peritoneal metastasis. After the tumor response in liver metastases, the PD-L1 expression in the peritoneal metastasis might have been up-regulated by increasing infiltration of CD8-positive T cells [17], which could have led to the delayed pseudoprogression documented in our case. Second, differences in the tumor growth rate could have led to the time lag between the two pseudoprogression events. The slower tumor growth of peritoneal metastasis than the liver metastases could have caused a time lag in the development of immune evasion. However, we could not prove the validity of our hypothesis by histopathology in this patient, because we could not obtain the patient’s consent to perform tumor biopsy after progression.

The presence of tumor-infiltrating lymphocytes (TILs) is an important factor in the immune responses of a tumor [18]. Although we did not quantitate the TILs histopathologically in this patient, we analyzed the leukocyte counts in the peripheral blood during the treatment. Some retrospective studies reported that the numbers of lymphocytes, monocytic myeloid-derived suppressor cells (MDSCs) and neutrophils in peripheral blood were associated with clinical outcome [19–22]. As shown in Fig. 4, during the treatment, the lymphocyte/monocyte ratio was elevated in this case. Increase in the number of lymphocytes in the blood suggests activation of the immune system. On the other hand, a stable number of monocytes indicates the stable presence of MDSCs, which inhibit cytotoxic T lymphocyte activity [20]. An increase in the count of MDSCs has been reported to be associated with tumor progression, poorer outcomes, and decreased effectiveness of immunotherapeutic strategies [19, 23], while a stable number of monocytes appears to be associated with good outcomes. Therefore, the lymphocyte/monocyte ratio in our patient suggests continued activation of the antitumor immune responses, which could have led to the serial pseudoprogression events.

According to the irRC, immune-related progressive disease is defined as “at least 25% increase in tumor burden compared with nadir (at any single time point) in two consecutive observations at least 4 weeks apart” [15]. There is no report or definition about serial pseudoprogression, and the emergence of a new lesion after early pseudoprogression is likely to be diagnosed as progressive disease. Our experience indicates that the development of a new lesion after early pseudoprogression might not always signify failure of disease control during treatment with nivolumab. Further accumulation of data is required to better understand the immune-related responses and for the confirmation of disease progression. In conclusion, we report a case of metastatic malignant melanoma that showed the unique response pattern of serial pseudoprogression during the course treatment with nivolumab. Development of a new lesion detected radiographically after early pseudoprogression might not always signify failure of disease control. More data are needed to understand immune-related responses to immunotherapy and for the confirmation of disease progression.

## Abbreviations

CT: computed tomography
HMB45: human melanin black 45
irRC: immune-related Response Criteria
LDH: lactate dehydrogenase
MDSCs: monocytic myeloid-derived suppressor cells
PD-1: programmed death 1
PET: positron emission tomography
RECIST: Response Evaluation Criteria In Solid Tumors
TILs: tumor-infiltrating lymphocytes
UICC: Unio Internationalis Contra Cancrum
